# Multiscale Geometric Characterization and Discrimination of Dermatoglyphs (Fingerprints) on Hardened Clay—A Novel Archaeological Application of the GelSight Max

**DOI:** 10.3390/ma18132939

**Published:** 2025-06-21

**Authors:** Katarzyna Peta, W. James Stemp, Tera Stocking, Richard Chen, George Love, Matthew A. Gleason, Brett A. Houk, Christopher A. Brown

**Affiliations:** 1 Institute of Mechanical Technology, Poznan University of Technology, Pl. Marii Skłodowskiej-Curie 5, 60-965 Poznań, Poland; 2 Department of Sociology, Anthropology and Criminology, Keene State College, 229 Main Street, Keene, NH 03435-3400, USA; jstemp@keene.edu; 3 Department of Anthropology, University of Kentucky, 150 Patterson Drive, Lexington, KY 40506-0027, USA; tstocking@uky.edu; 4 Surface Metrology Laboratory, Mechanical and Materials Engineering Department, Worcester Polytechnic Institute, 100 Institute Road, Worcester, MA 01609-2280, USA; rchen8@wpi.edu (R.C.); glove2@wpi.edu (G.L.); magleason@wpi.edu (M.A.G.); brown@wpi.edu (C.A.B.); 5 Department of Sociology, Anthropology, and Social Work, Texas Tech University, 1101 Boston Ave., #158, Lubbock, TX 79409-1012, USA; brett.houk@ttu.edu

**Keywords:** GelSight Max, elastomer tactile sensor, pottery, fingerprint, surface metrology

## Abstract

A relatively new GelSight Max measurement instrument was applied to the microtopographies of experimental hardened clay surfaces, both with and without fingerprint (dermatoglyph) impressions, and the surface of an archaeological pottery handle fragment with a preserved fingerprint (paleodermatoglyph). The experimental clay surfaces were documented in order to determine the instrument’s ability to capture these surfaces in three-dimensions by imprinting them onto an elastomeric tactile membrane. Fingerprints on the experimental hardened clay and the archaeological pottery fragment were mathematically documented to test this instrument’s ability to capture these impressions. The surface texture measurements of the hardened clay and the pottery fragment were mathematically compared using conventional topographic characterization parameters (height and hybrid), fractal dimensions (Das) with associated coefficients of determination (R^2^), and multiscalar geometric characterization parameters, particularly relative area (Srel), area-scale complexity (Asfc), relative length (RL), and length-scale complexity (Lsfc). The surfaces of the experimental hardened clay with and without fingerprints and the archaeological pottery handle fragment with a fingerprint can be discriminated using some conventional height parameters, as well as some multiscale geometric topographic characterization parameters. Specifically, relative area (Srel), area-scale complexity (Asfc), relative length (RL), and length-scale complexity (Lsfc) could all discriminate between the hardened clay block with and without fingerprints and the fingerprint on the archaeological pottery handle fragment at different scales of measurement. Mean square ratios (MSRs) above 90% and 95% confidence levels indicated that the discrimination of these multiscale geometric characterizations was significant. In sum, the GelSight Max has the potential to be a valuable instrument for archaeologists studying pottery and fingerprints.

## 1. Introduction

### 1.1. Purpose and Scope of the Research

Fingerprints (i.e., dermatoglyphs) are unique features of primate and koala hands that provide superior grip by interlocking with rough surfaces, channeling water, and improving tactile sensitivity [1]. It is argued that no two people’s fingerprints are identical and thus fingerprints are extremely valuable physical characteristics for identifying individual human beings [2,3,4,5]. Unsurprisingly, the ability to identify individuals based on fingerprints has been of interest to archaeologists given the potential to learn about individuals whose fingerprints (i.e., paleodermatoglyphs) may have been preserved in various media in the past, notably clays used in pottery production. Although there are previous studies of fingerprints preserved on clay pottery recovered from archaeological sites, to our knowledge, no one has attempted to quantitatively document and discriminate the two- and three-dimensional structure of fingerprints preserved on archaeological pottery surfaces using both topographic height and hybrid parameters and multiscale geometric analysis.

A relatively new and portable GelSight Max measurement instrument that uses an elastomeric tactile sensor to conform to surfaces and capture the texture of the measured surfaces has previously been used on stone tools to document different raw material types and microwear [6]. Based on the results of that experiment to determine potential archaeological applications for stone artifacts, in this experiment, we used the instrument to measure topographies for the mathematical documentation of experimental clay surfaces, with and without fingerprints, and on an archaeological pottery handle fragment with a preserved fingerprint.

In order to discriminate the surface structures of the hardened clay and the fingerprints on them, conventional height (Sa, Sq, Ssk, Sku, Sp, Sv, and Sz) and hybrid (Sdq and Sdr) topographic characterization parameters, fractal dimensions (Das) with associated coefficients of determination (R^2^), and multiscale geometric characterization parameters, specifically relative area (Srel), area-scale complexity (Asfc), relative length (RL), and length-scale complexity (Lsfc), were calculated from the surface data measured using the instrument (Table 1). Results indicate the successful discrimination of the surfaces in many, but not all, cases based on these parameters and multiscale geometric characterizations.

### 1.2. Fingerprint Structure and Documentation

Fingerprint patterns consist of three main types: arches, loops, and whorls, which include more precise internal distinctions and singular points, such as ulnar loops, radial loops, cores, deltas, and triradii. Fingerprints also possess defect patterns, known as minutiae, such as dislocations, island ridges, and incipient ridges [2,7,8,9,10]. This means that there is huge variation in the fingerprint patterns of individuals. The science of documenting fingerprints for the purposes of human identification emerged in the late 19th century; however, the value and study of fingerprints as identifiers is much older [2,9,11,12]. Although numerous techniques have been developed to identify individuals or general categories of people in terms of age, sex, and ethnicity, based primarily on ridge matching and minutiae, two common approaches include methods to determine fingerprint ridge density and ridge breadth (e.g., [3,4,13,14,15,16,17,18,19,20,21,22]).

### 1.3. Fingerprint Analysis on Archaeological Pottery: A Brief Summary

Fingerprints recovered from archaeological material (i.e., paleodermatoglyphs) can aid researchers in the identification of the age and sex of craft producers and are one of the only direct indicators of potter identity [16,23,24,25,26]. Ceramic materials, in particular, preserve fingerprints well due to the plasticity of the clay and the ceramic manufacturing process, which includes shaping, molding, and handling wet clay. After the ceramic is dried and fired, the clay hardens which results in a chemically stable material, allowing the fingerprints to be preserved [16]. These fingerprints can be sampled using high resolution photography, 3D scanning, or molded impressions and then measured using mean ridge breadth (MRB) and mean ridge density (MRD) to ascertain both the sex and age of producers [25,26]. Although dermatoglyph research has been embraced by forensic anthropology and law enforcement, the study has been largely dismissed by archaeologists as a means of studying craft production [27].

The first published study on fingerprints on pottery was conducted in 1880 by Henry Faulds [28], who identified hand furrows on Japanese ceramics. Faulds (1880) [28] argued that fingerprints were an acceptable way of studying ancient ceramic producers and advocated for fingerprints in the identification of criminals. There was a considerable length of time before fingerprinting once again appeared in archaeological literature. Harold Cummins [29] published *Ancient Finger Prints in Clay* in 1941 that, while a thorough accounting of the fingerprints found on Grecian vessels, failed to bring increased enthusiasm from other archaeologists [27]. A single study in 1980 by Paul Åström and Sven A. Eriksson [23] furthered the study of paleodermatoglyphs by explaining their use as a source of data to the discipline. This early work focused on using forensic methodologies of collecting and comparing fingerprints. Åström and Eriksson (1980) [23] determined that three points of comparison were necessary for comparing ancient potter fingerprints and for the identification of specific individual potters.

In the 1990s and 2000s, research focused on other measurements of fingerprints, including mean ridge-furrow pairs ridge breadth (MPRB) and mean ridge density (MRD), that can assess age and sex but cannot identify specific individuals. MPRB is measured perpendicularly from the beginning of one ridge, across the furrow, to the beginning of the next ridge and then divided by the number of ridge pairs. MRD is simply the number of ridges within a predetermined space [25]. Kamp et al.’s (1999) [24] seminal work on fingerprints and childhood presented archaeologists with the possibility of shedding light on both age and gender in the archaeological record. The study used fingerprints found on figurines and a regression formula using MPRB to determine the age of the maker and potential situated learning methods. Following this study, Králík and Novotný (2003) [16] applied forensic techniques of ridge breadth and density analysis to archaeological fingerprints, finding that it was possible to reliably determine sex from these artifacts. This type of analysis and Kamp’s regression formula have been applied in a small number of papers in various cultural contexts, including the American Puebloan Southwest, Bronze Age Israel, and Teotihuacan, which used the paleodermatoglyph data to examine the identity of potters, social inequalities, and potential sexual divisions of labor (e.g., [25,27,30,31,32,33,34,35]).

More recent studies combine both ridge breadth and ridge density measurements into one matrix. Kent Fowler et al. (2019) [25] established an identification framework which builds on previous studies by incorporating a modified version of the regression formula proposed by Kamp et al. (1999) [24] and the MRD established by Králík and Novotný (2003) [16]. The matrix they propose allows for the identification of adult males, adult females, adolescent males, adolescent females, and children, which is a more precise categorization of potter identity than studies that came before [25,26]. This identification matrix has also been used in the most recent fingerprint study by Dyowe Roig et al. (2023) [36].

Several methods have been employed for identifying and measuring paleodermatoglyphs on archaeological material. Most early studies use a directed light source at a 45-degree angle to the sherd’s surface to identify the print. Then, ceramic casts, photographs, microscope images, micro-CT scanning, or 3D scanning are employed for the identification of prints, followed by digital measurement tools for assessing MRB and MRD for recording the print itself [16,23,24,25,26,27,31,36,37]. Moreover, Bayesian models have also been used to determine the demographics of potters in ancient societies [38,39,40]. These methods provide different types of two-dimensional (2D) and/or three-dimensional (3D) reproductions of fingerprint impressions with variable degrees of reliability based on the specific technology used; however, the data generated from these surface reproductions is typically only used for the mathematical characterization of print features on a single plane (x- and y-axes), such as MRB and MRD, with no calculation of heights (z-axis). As such, although important data about fingerprint ridge and furrow spacing and the number of ridges within a certain area can be generated, combinations of these approaches have not been used to calculate the surface roughness or texture of fingerprints in hardened clay. Given that ancient fingerprints have been underutilized by archaeologists, there is currently no unified methodology for their identification.

### 1.4. GelSight and Fingerprints

To our knowledge, there is only one study that has employed GelSight technology to document human fingerprints. In the study by Beatty et al. (2024) [41], both a Sensofar S Neox confocal/focus variation microscope and a GelSight Mobile 2 instrument were used to quantitatively document the surfaces of clear epoxy resin casts (Epokwik, Buehler) made from the fingerprints of four cadavers. The surface data generated from the scans taken using the confocal Sensofar S Neox microscope and the GelSight Mobile 2 instrument were compared using multiple SSFA (scale-sensitive fractal analysis) parameters, specifically maximum relative distance (Y max), smooth–rough crossover threshold (SRC threshold), length-scale complexity (Lsfc), and fractal dimensions (Das). Results indicated that the SSFA parameters could statistically distinguish the fingerprints of some individuals from those of others, but not in all instances. There was also variation in terms of the statistically significant distinction of fingerprints based on SSFA parameters for the Sensofar versus GelSight surface data. Overall, Beatty et al. (2024) [41] observed that the GelSight surface data seemed to provide more variables that could differentiate the fingerprints of the four individuals than the Sensofar.

### 1.5. The GelSight Max Instrument

**Figure 1:**
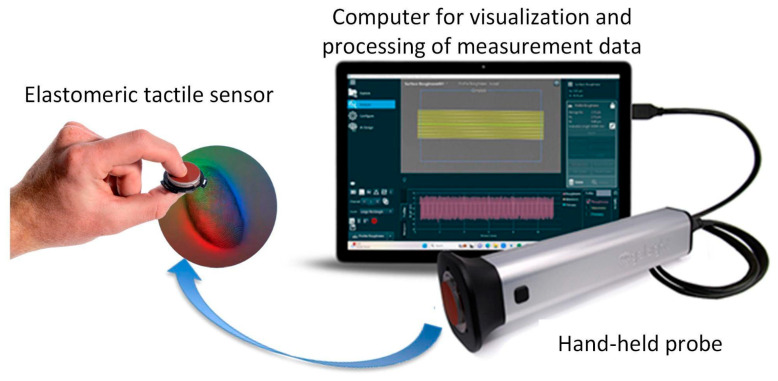
The GelSight Max (figure compiled from photos from the official GelSight manufacturer’s website—https://www.gelsight.com).

GelSight is a measurement system with an elastomeric tactile sensor for the direct acquisition of topographic heights (z) at specific locations (x, y) for characterizing the three-dimensional geometry of a surface [42,43,44,45]. This instrument uses a hand-held probe with a thermoplastic polyurethane gel pad with a deformable reflective membrane illuminated from behind by LEDs. When pressed onto an object, the instrument membrane interfaces with the surface to provide a constant contrast during topographic measurements with light reflection. The impression of the surface on the membrane forms a replica of the object’s texture, with the images recorded by the camera. The three-dimensional height map (z) in the coordinate (x, y) system is calculated by the photometric reflectance transformation imaging algorithm and is created based on the angles of inclination of the direction and brightness of the pixels. The captured surface images are processed in dedicated GelSight software (https://www.gelsight.com). The manufacturer’s specifications are provided in Table 2. In this experiment, the GelSight Max instrument was adopted to measure the surfaces of the hardened clay and archaeological pottery (Figure 1).

### 1.6. Scale-Sensitive Fractal Analysis

As discussed by Stemp et al. (2016) [46], over the last two decades, the mathematical documentation of surface textures preserved on materials excavated by paleoanthropologists and archaeologists has increased substantially. In particular, mathematical descriptions of irregular surfaces at multiple observation scales, using scale-sensitive fractal analysis (also called multiscale geometric analysis), have been important for analyzing stone, bone, and tooth materials recovered from archaeological and paleoanthropological sites (e.g., [6,47,48,49,50,51,52,53,54,55,56,57,58,59,60,61,62,63,64,65,66,67,68,69]).

**Figure 2:**
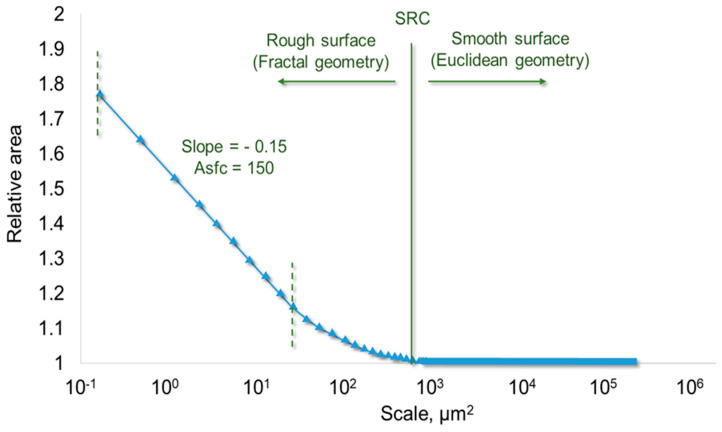
Relative area over scale range. The slope is used in area-scale complexity (Asfc) calculations. A curve deviating from a relative area of one is associated with the SRC, which is the scale of transition from a smooth surface described by regular Euclidean geometries to rough surfaces characterized by irregular fractal geometries.

Scale-sensitive analyses originated from Mandelbrot’s work on fractal shapes [70,71], which were applied years later to the characterization of surfaces (e.g., [72,73]). Topographic measurements can be analyzed at multiple, different observation scales, ranging from as small as the sampling interval to the overall size of the entire measurement distance or area. Fine scale ranges can be related to surface roughness, although the designer is responsible for determining the cut-offs for the roughness scales [74,75]. The S and R [74,75] topographic parameters in this study were calculated and related to fine scales. This is due to the relatively small measurement regions, the observation of the surface at fine scales, and the abandonment of the use of data filtering, including both L and S cut-off filters. Although often used colloquially, “roughness” is related to the parameters of topographic surface characterization at a limited scale in surface engineering. When dealing with measured topographies in terms of “roughness”, the smooth–rough crossover (SRC) can be valuable. The SRC determines the boundary (i.e., specific scale) where the surface is represented by regular Euclidean geometries related to coarser scales as opposed to finer scales. At finer scales below the SRC, the surface appears rough (and is more complex) and is better described using scale-sensitive fractal analysis [76] (Figure 2).

For our study, we used relative area (Srel), from which we calculated area-scale complexity (Asfc), and relative length (RL), from which we calculated length-scale complexity (Lsfc) [74,75], to describe the surface microgeometries of the experimental hardened clay blocks with and without fingerprints and the archaeological pottery handle fragment with a fingerprint. We selected these four parameters given their successful application in a previous study documenting stone tool surfaces using the GelSight Max instrument [6]. Srel is the calculated area (CA) of a measured surface at a certain scale (s) divided by the nominal area (NA) of that same surface, whereas RL is the calculated length (CL) of a measured profile at a certain scale (s) divided by the nominal length (NL) of that profile (1) [72].

RL(s) = CL(s)/NL   Srel(s) = CA(s)/NA (1)

**Figure 3:**
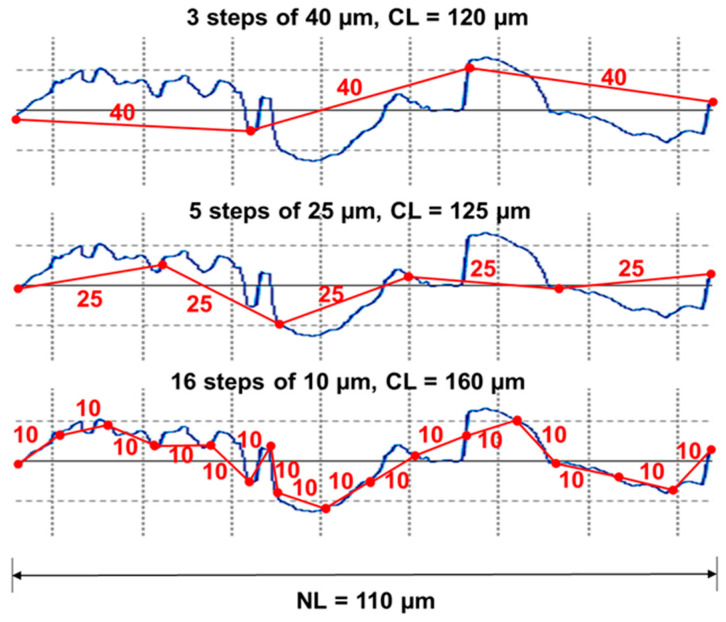
Example of length-scale analysis for three different calculated lengths (CLs) over a nominal length (NL) of 110 µm.

**Figure 4:**
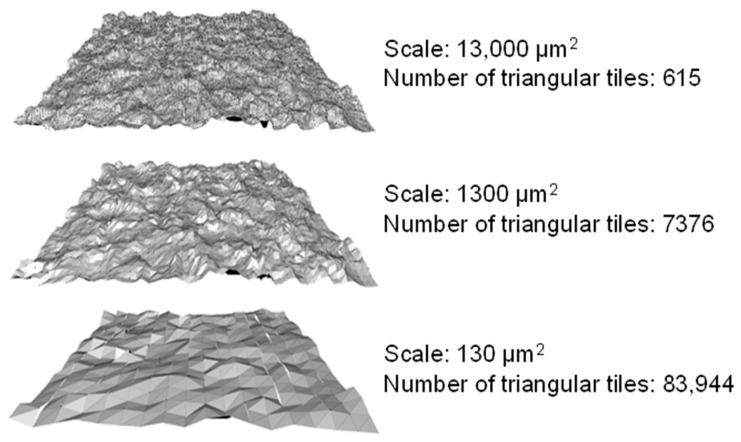
Example of area-scale analysis for three different calculated areas (CAs) over a nominal area (NA) of 2800 × 2800 µm^2^.

Multiscale geometric analysis represents CAs and CLs on a surface covered with tile triangles (area) and line segments (length) relative to NAs and NLs, as presented in Figure 3 and Figure 4.

The CAs refer to the number of tile triangles at a given scale required to cover a specified NA, whereas the CLs refer to the number of linear segments at a given scale required to cover a specified NL. Srel/RL are associated with the slope or angle of the tile triangles/line segments as weighted averages of the reciprocal of the cosine the tile triangle/line segment creates with the datum/horizontal plane for said surface (*s*, *S*) or profile (*p*, *P*). Angle θ is between the tile triangles or linear segments.

(2) $$RL\left(s\right)=\sum_{i=1}^{N}\frac{1}{\cos\theta_{i}}\frac{p_{i}}{P}\;\;\;Srel\left(s\right)=\sum_{i=1}^{N}\frac{1}{\cos\theta_{i}}\frac{s_{i}}{S}$$

Srel and RL, ideally, are largest at the finest scales and decrease at coarser scales. Srel and RL values that are >1 indicate that the analyzed surfaces can be described by Equation (2). When the Srel or RL is larger than one at the coarsest scales, it is recommended to reduce the minimum value of Srel or RL over the entire range of values to obtain a minimum value of one. Complexity is directly independent of the Srel and RL values but is related to their changes. Both Asfc and Lsfc are calculated from the log-log plot of Srel and RL at their corresponding scales, as −1000 × slope. Any changes in Srel and RL are related to the negative slopes of the logarithmic plot of Srels or RLs versus scales. The surface complexity is greater as Asfc and Lsfc increase [6,72,76,77]. The multiscale geometric parameters can be considered filtered characteristics. Srel and RL refer to low-pass characteristics, and Asfc and Lsfc to band-pass filter characteristics. Larger surface features affect Srel and RL at finer scales, while Asfc and Lsfc are associated with a narrow band of observation scales.

Another parameter calculated from the Srel and scale graph is the fractal dimension (Das), which is defined as 2–2 × slope. The coefficient of determination R^2^ is closely related to Das and demonstrates that the surface complexity is consistent in a given region of scales.

## 2. Materials and Methods

### 2.1. Materials

**Figure 5:**
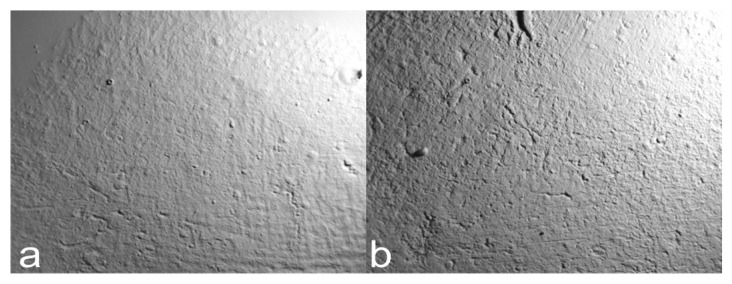
Examples of photosimulations of the unused surfaces of the hardened clay produced experimentally based on measurements using the GelSight Max instrument: (**a**) Block 1 [Pottery surface (scan 1)] and (**b**) Block 2 [Pottery surface (scan 2)].

**Figure 6:**
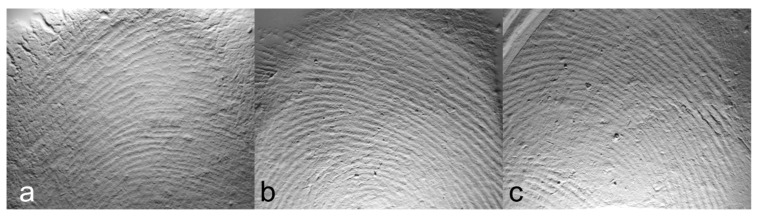
Examples of photosimulations of the right thumb impressions experimentally produced in the hardened clay based on measurements using the GelSight Max instrument: (**a**) Individual 1—right thumbprint 1 [Fingerprint (scan 1)], (**b**) Individual 2—right thumbprint 1a [Fingerprint (scan 2)], and (**c**) Individual 2—right thumbprint 1b [Fingerprint (scan 3)].

**Figure 7:**
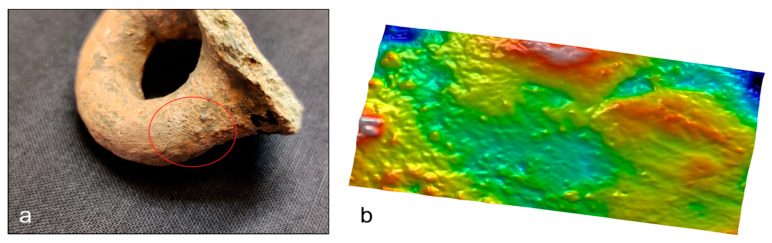
Pottery handle fragment from an unprovenanced collection from excavations by Matthew Stirling conducted during the early 1940s showing (**a**) the location of the fingerprint (red circle) and (**b**) a 3D height map of the fingerprint from the elastomer gel impression [Handle (scan 1)] showing the ridges and furrows.

**Figure 8:**
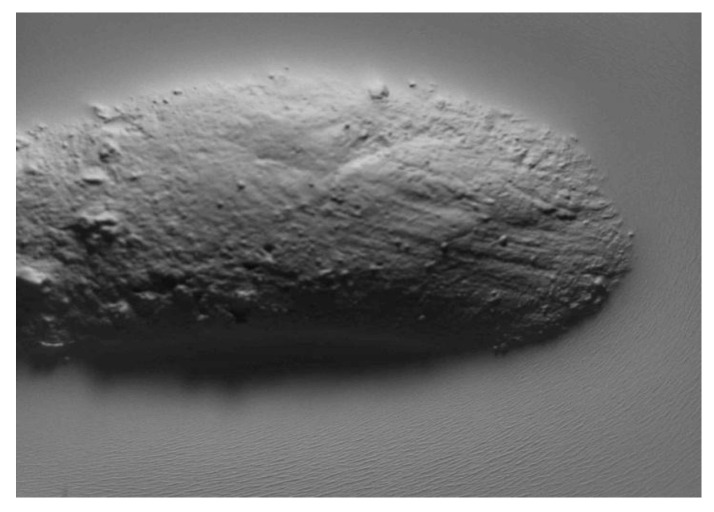
An example of the photosimulation of the fingerprint impression on the surface of the archaeological pottery handle fragment based on measurements using the GelSight Max instrument for Pottery handle fragment 1—print 1a [Handle (scan 1)].

We tested the GelSight Max instrument on experimental and archaeological hardened clay to determine its ability to document fingerprint impressions, specifically the surfaces of two blocks of hardened clay (Block 1 and Block 2) without fingerprints (Figure 5), the surfaces of three blocks of hardened clay with one fingerprint (Individual 1—right thumbprint 1; Individual 2—right thumbprint 1a; Individual 2—right thumbprint 1b) (Figure 6), and the surface of the handle of a pottery fragment with a preserved fingerprint from an unprovenanced collection from excavations conducted during the early 1940s by Matthew Stirling (Pottery handle fragment 1—prints 1a-c) (Figure 7a,b, and Figure 8) (Table 3).

### 2.2. Surface Measurement and Analysis

**Figure 9:**
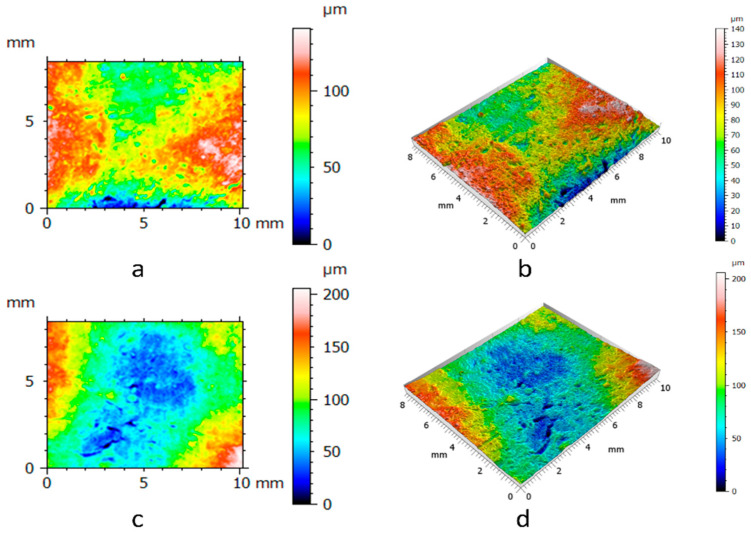
The 2D and 3D height maps of the hardened clay blocks without fingerprint impressions of (**a**) Pottery surface (scan1) (2D), (**b**) Pottery surface (scan 1) (3D), (**c**) Pottery surface (scan 2) (2D), and (**d**) Pottery surface (scan 2) (3D).

**Figure 10:**
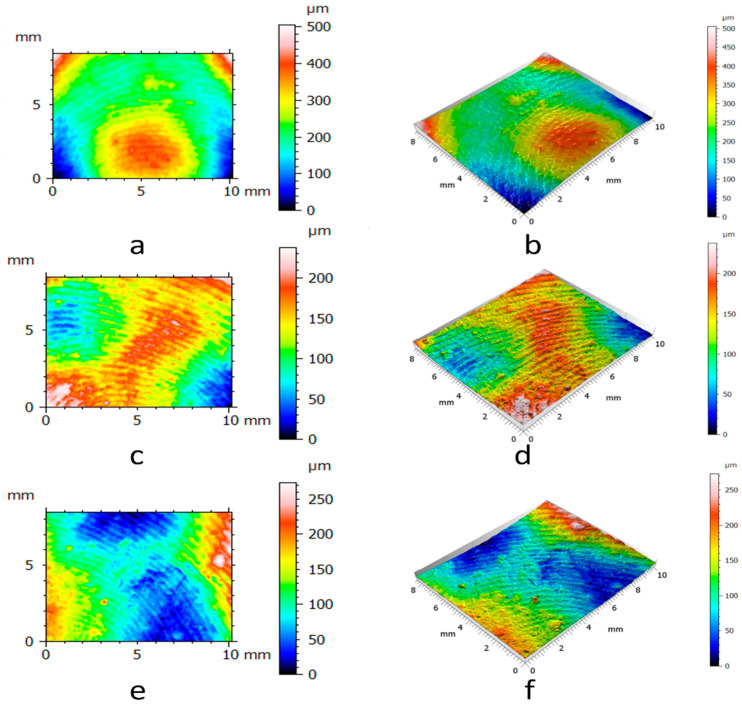
The 2D and 3D height maps of the hardened clay blocks with fingerprint impressions of (**a**) Fingerprint (scan 1) (2D), (**b**) Fingerprint (scan 1) (3D), (**c**) Fingerprint (scan 2) (2D), (**d**) Fingerprint (scan 2) (3D), (**e**) Fingerprint (scan 3) (2D), and (**f**) Fingerprint (scan 3) (3D).

**Figure 11:**
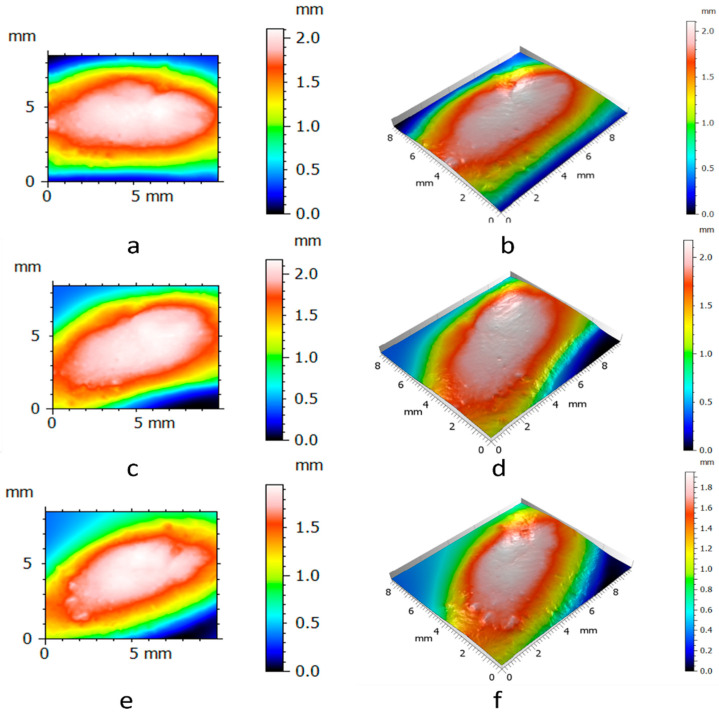
The 2D and 3D height maps of the fingerprint of the archaeological pottery handle fragment impressions of (**a**) Handle (scan 1) (2D), (**b**) Handle (scan 1) (3D), (**c**) Handle (scan 2) (2D), (**d**) Handle (scan 2) (3D), (**e**) Handle (scan 3) (2D), and (**f**) Handle (scan 3) (3D).

To measure the surfaces of the clay blocks and the pottery handle fragment, we pressed the GelSight Max’s elastomeric gel membrane into the measured objects to capture topographic images. From the tactile sensor’s standard field of view (14.6 mm × 8.3 mm), we selected smaller areas of the surfaces (10 mm × 8.3 mm) from the experimental hardened clay blocks with and without fingerprints (Figure 9 and Figure 10) and the archaeological pottery handle fragment with the fingerprint (Figure 11). The measurements faithfully captured the surfaces and no outliers or non-measured points were identified. Surface renderings provided complete source data for processing in topographic analysis software, including their two- and three-dimensional representations. A set of height points at specific x and y locations (height maps) allowed for further calculations of conventional and multiscale geometric topographic parameters.

For each of the two experimental hardened clay blocks without fingerprints and the three experimental clay blocks with fingerprints, we took one impression using the gel membrane to produce the 2D and 3D height maps. However, to better capture the surface microtopography of the fingerprint on the archaeological pottery handle fragment, we took three impressions in the gel membrane to produce the 2D and 3D height maps (Figure 9, Figure 10 and Figure 11). These height maps were used to determine conventional topographic characterization parameters (height and hybrid), Das with associated R^2^, and multiscale geometric characterizations parameters, specifically Srel and Asfc for each of the surfaces. In order to establish the statistical significance of Srel and Asfc for the impressions of the two experimental hardened clay blocks without fingerprints [Pottery surface (scan 1) and Pottery surface (scan 2)], the three experimental clay blocks with fingerprints [Fingerprint (scan 1), Fingerprint (scan 2), and Fingerprint (scan 3)], and the location on the pottery handle fragment with the fingerprint [Handle (scan 1), Handle (scan 2), and Handle (scan 3)], we calculated mean square ratios (MSRs) with the confidence levels set at 90% and 95%.

**Figure 12:**
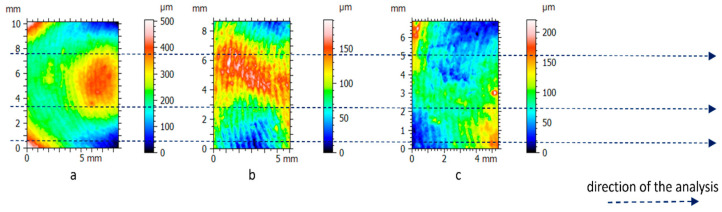
The 2D height maps of the hardened clay blocks with fingerprint impressions of (**a**) Fingerprint (scan 1), (**b**) Fingerprint (scan 2), and (**c**) Fingerprint (scan 3) aligned for profile data collection oriented perpendicularly to the fingerprint ridges and furrows.

For each of the height maps for the three experimental clay blocks with fingerprints and the location on the pottery handle fragment with the fingerprint, we extracted three profiles oriented perpendicularly to fingerprint ridges and furrows (e.g., Figure 12) to calculate average RL and Lsfc. We also extracted three profiles from the height maps for the two experimental hardened clay blocks without fingerprints for the purposes of calculating the average RL and Lsfc. We confirmed the statistical significance of RL and Lsfc for the two experimental hardened clay blocks without fingerprints, the three experimental clay blocks with fingerprints, and the location on the handle fragment with the fingerprint on the basis of MSRs calculated using confidence levels of 90% and 95%. A reorientation of the height maps to align the three profiles perpendicularly to the fingerprint ridges and furrows resulted in extracted surface sampling areas of slightly different sizes.

We processed the topographic data from the surface measurements with DigitalSurf’s MountainsMap version 9 software and mathematically documented the different experimental and archaeological hardened clay surface impressions using Sa, Sq, Ssk, Sku, Sp, Sv, Sz, Sdq, Sdr, Srel, Asfc, RL, and Lsfc parameters [74,75] over a range of areal scales (35.4 µm^2^ to 35,424,900 µm^2^) and length scales (10.3 µm to 8472.2 µm). We preprocessed the topographic data by surface leveling using the least-squares, material ratio-based thresholding, and removing any outliers.

## 3. Results

An analysis of the surface data derived from the measured regions on the experimental hardened clay blocks with and without fingerprints and the archaeological pottery handle fragment with the fingerprint provided interesting results. We used the height maps produced using the GelSight Max instrument to calculate conventional height and hybrid parameters, specifically the first four statistical moments of heights (Sa, Sq, Sku, and Ssk), extreme heights (Sv, Sz, and Sp), and hybrid parameters (Sdq and Sdr) (Table 4). We noted significant differences for most parameters calculated for each of the individual surfaces of the hardened clay blocks with and without fingerprints and the archaeological pottery handle fragment with the fingerprint. However, for some parameters, no significant differences existed. We determined that, based on the means of these parameters coupled with their standard deviations, the fingerprint on the pottery handle fragment could be discriminated from the means with standard deviations for both the hardened clay blocks without fingerprints and those with fingerprints. But our data demonstrated that the surfaces of the hardened clay blocks without fingerprints and those with fingerprints could not be mathematically discriminated based on the means with standard deviations for the Ssk, Sku, Sp, and Sz parameters.

In addition to the height and hybrid parameters, we calculated R^2^ of Das from the instrument’s height maps for both the experimental and archaeological hardened clay surfaces. Our calculations of the means with standard deviations for the R^2^ of the surfaces of the hardened clay blocks with and without fingerprints and the fingerprint on the pottery handle fragment demonstrated that none of the surfaces could be discriminated. However, the means with standard deviations calculated for Das could discriminate the surfaces of the blocks with fingerprints from those without. Conversely, for Das, we found that neither the mean with standard deviation for the surfaces of the hardened clay blocks with fingerprints nor the mean for those without prints could be discriminated from the mean with standard deviation for the fingerprint on the surface of the pottery handle fragment (Handle (scans 1–3)) (Table 5).

**Figure 13:**
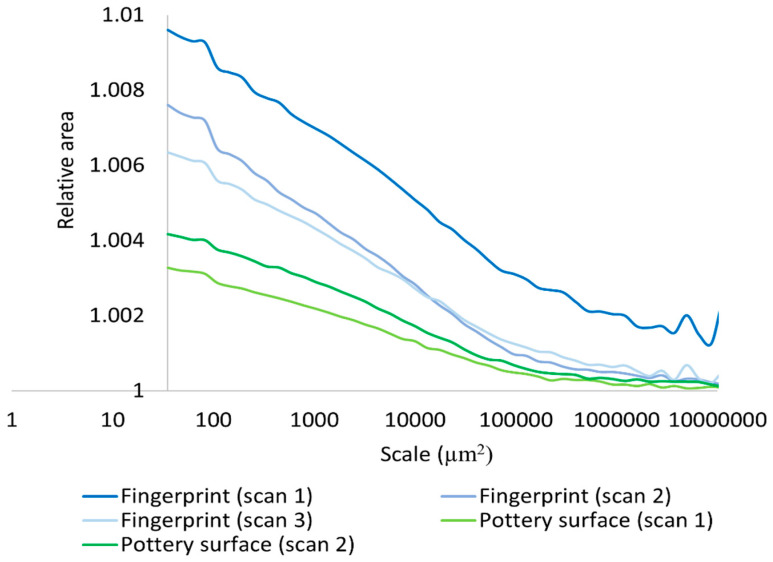
Srel for the experimental hardened clay blocks with [Fingerprint (scans 1–3)] and without [Pottery surface (scans 1–2)] fingerprints.

**Figure 14:**
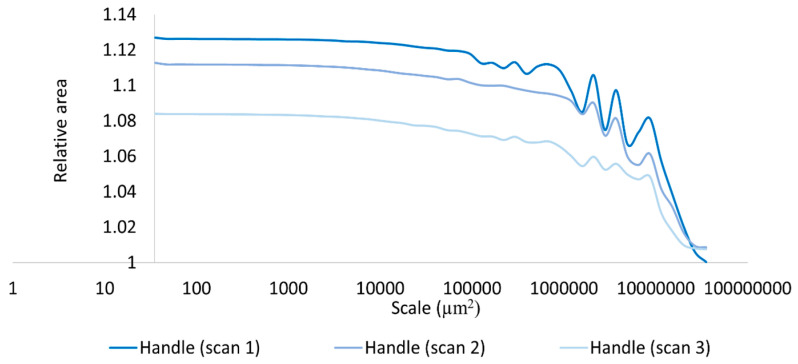
Srel for the fingerprint on the pottery handle fragment [Handle (scans 1–3)].

We also performed a multiscale characterization of the surface properties using Srel, RL, Asfc, and Lsfc. Calculation of Srel were possible for the experimental hardened clay block surfaces with and without fingerprints and the surface with the fingerprint on the archaeological pottery handle fragment over multiple scales (Figure 13). A decrease in the scale of calculation resulted in an increase in Srel for all measured surfaces. Nevertheless, our results revealed that the three measurements on the pottery handle fragment with the fingerprint began to level off beginning around 1000 µm^2^ (Figure 14). This is because the relative minima at large scales are due to periodicity (persistent spatial frequencies) in the topographic data, as should be expected for fingerprints. The minima occur at integer multiples of the dominant wavelength (the inverse of spatial frequency), which are due to aliasing with the tiling elements. The values greater than one at the highest scales indicate that the surface is sloped at those scales, or that the largest scale is above the SRC. Our results demonstrated that Srel calculated from the surfaces both with and without fingerprints on the hardened clay blocks and the fingerprint on the pottery handle fragment varied over multiple areal scales.

**Figure 15:**
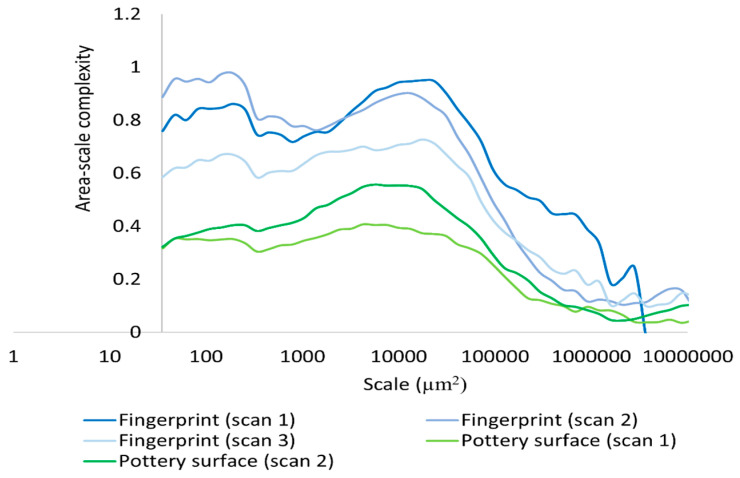
Asfc for the experimental hardened clay blocks with [Fingerprint (scans 1–3)] and without [Pottery surface (scans 1–2)] fingerprints.

**Figure 16:**
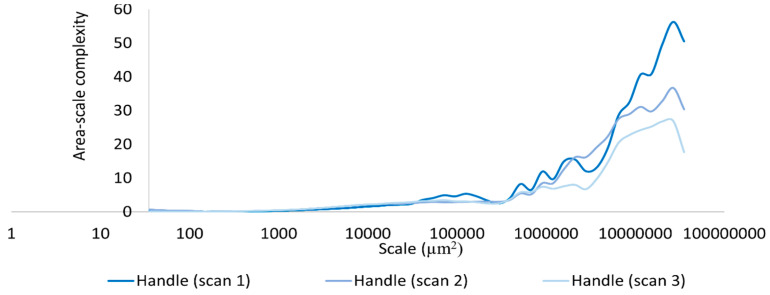
Asfc for the fingerprint on the pottery handle fragment [Handle (scans 1–3)].

**Figure 17:**
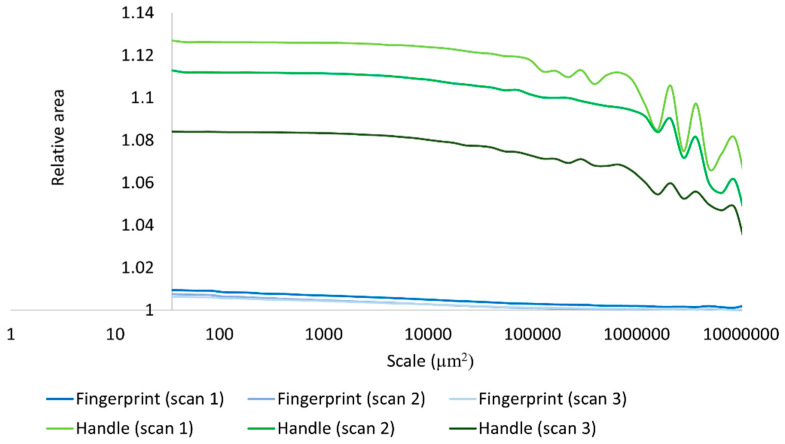
Srel for the experimental hardened clay blocks with fingerprints [Fingerprint (scans 1–3)] and the fingerprint on the pottery handle fragment [Handles (scans 1–3)].

**Figure 18:**
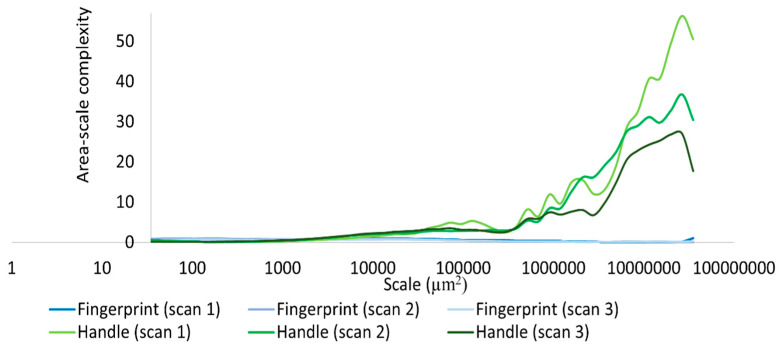
Asfc for the experimental hardened clay blocks with fingerprints [Fingerprint (scans 1–3)] and the fingerprint on the pottery handle fragment [Handle (scans 1–3)].

**Figure 19:**
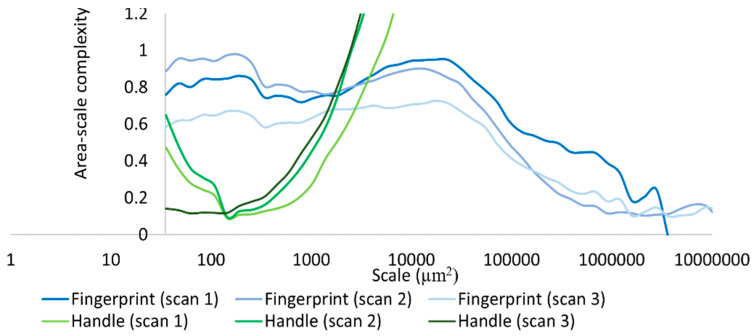
Asfc for the experimental hardened clay blocks with fingerprints [Fingerprint (scans 1–3)] and the fingerprint on the pottery handle fragment [Handle (scans 1–3)]. Note the reduction in y-axis scale.

Based on our surface data, we could calculate Asfc over multiple areal scales and demonstrate differences in Asfc over these scales for all measurements on the hardened clay blocks with and without fingerprints and the fingerprint on the pottery handle fragment. In particular, Asfc was higher for the hardened clay blocks with the fingerprints compared to the hardened clay blocks without them. (Figure 15). In our results, Asfc leveled off for the graph plots for the pottery handle fragment with the fingerprint around 1000 µm^2^ (Figure 16). This observation was similar to our graphs for Srel. The surfaces on the hardened clay blocks with fingerprints demonstrated consistently lower Srel over multiple areal scales than the pottery handle fragment with the fingerprint, indicating less surface complexity on the former (Figure 17). Moreover, Asfc for the pottery handle fragment with the fingerprint was consistently higher at coarse scales above 1000 µm^2^ than Asfc for the hardened clay blocks with fingerprints (Figure 18); however, the Asfc data became difficult to graph with a large y-axis scale. By reducing the y-axis scale for the Asfc graph, we could more easily observe the differences between the hardened clay blocks with fingerprints and the pottery handle fragment with the fingerprint at fine scales below 1000 µm^2^. At the reduced y-axis scale, the pottery handle fragment with the fingerprint presented lower Asfc than the hardened clay blocks with fingerprints (Figure 19).

**Figure 20:**
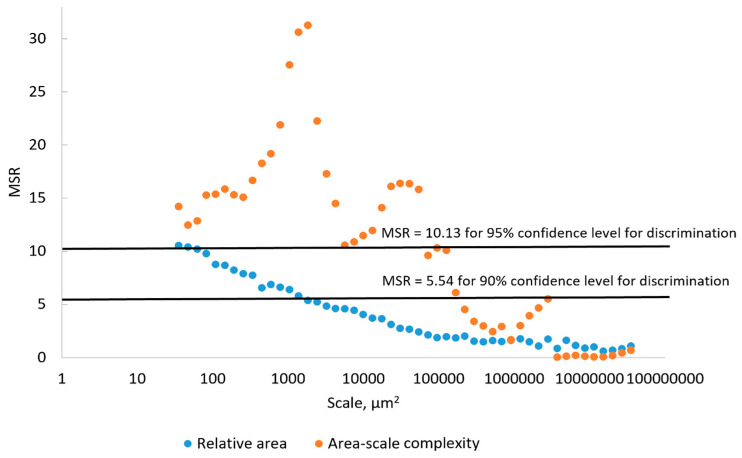
MSRs of mean Srel and Asfc for discriminating the hardened clay blocks with [Fingerprint (scans 1–3)] and without [Pottery surface (scans 1–2)] fingerprints. The two horizontal lines denote the minimum MSR of 90% and 95% confidence levels where discrimination is possible.

In order to determine the degree to which the differences in mean Srel and mean Asfc documented on the surfaces of the hardened clay blocks without fingerprints and those with fingerprints were statistically significant, we calculated their MSRs at the 90% and 95% confidence levels (Figure 20). Based on mean Srel, our results indicated that the discrimination of the surfaces of the hardened clay blocks with and without fingerprints was not possible for either the 90% or 95% confidence levels at the coarse scales above 1000 µm^2^ and the discrimination of the surfaces was just possible beginning around 50 µm^2^ at the 95% confidence level. Based on the mean Asfc, our results showed that MSRs for the surfaces of the hardened clay blocks without fingerprints and those with fingerprints could not be discriminated at coarse scales above 100,000 µm^2^, but the mean Asfc of these different surfaces could be discriminated below 100,000 µm^2^ at both the 90% and 95% confidence levels.

**Figure 21:**
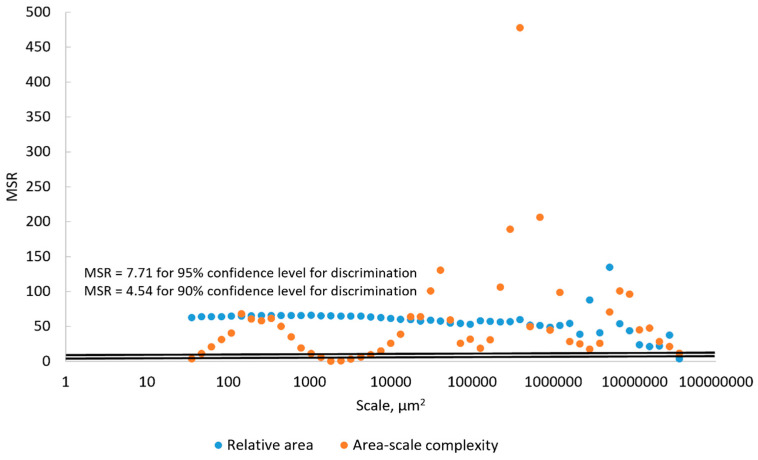
MSRs of mean Srel and Asfc for discriminating the experimental hardened clay blocks with fingerprints [Fingerprint (scans 1–3)] and the fingerprint on the pottery handle fragment [Handle (scans 1–3)]. The two horizontal lines denote the minimum MSR of 90% and 95% confidence levels where discrimination is possible.

For the MSRs of mean Srel and mean Asfc for the discrimination of the experimental hardened clay blocks with fingerprints and the fingerprint on the pottery handle fragment, our results were different (Figure 21). Based on our results, the mean Srel of the fingerprints on the hardened clay blocks and the fingerprint on the pottery handle fragment could be discriminated at almost any scale above both the 90% and 95% confidence levels. In contrast, MSRs for the mean Asfc of the surfaces of the hardened clay blocks with fingerprints and the fingerprint on the pottery handle fragment indicated that discrimination was not possible at scales from 1000 µm^2^ to 8000 µm^2^, but was possible at all other scales above the 90% and 95% levels.

**Figure 22:**
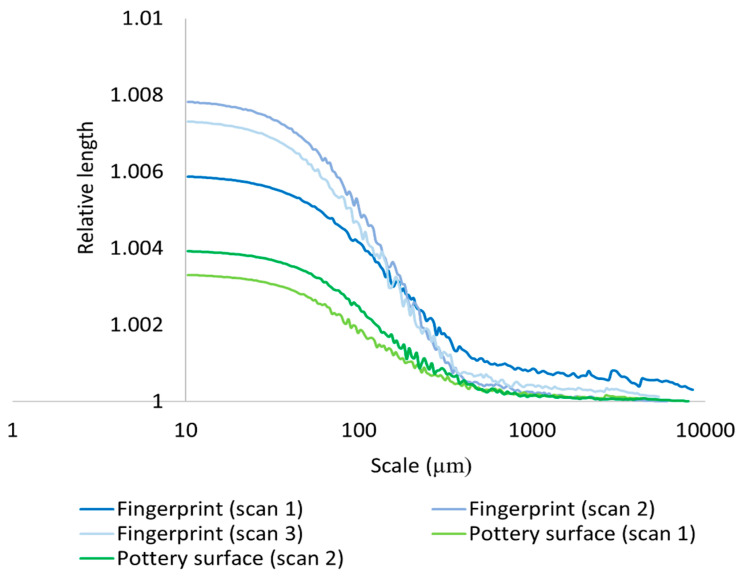
Mean RL for the experimental hardened clay blocks with [Fingerprint (scans 1–3)] and without [Pottery surface (scans 1–2)] fingerprints based on profiles perpendicular to the orientation of the fingerprint ridges and furrows.

**Figure 23:**
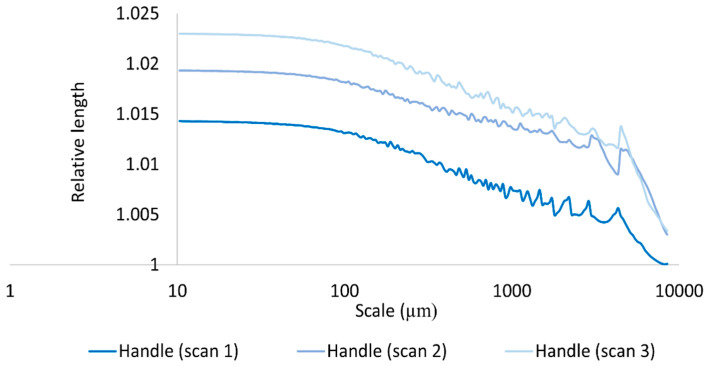
Mean RL for the fingerprint on the pottery handle fragment [Handle (scans 1–3)] based on profiles perpendicular to the orientation of the fingerprint ridges and furrows.

**Figure 24:**
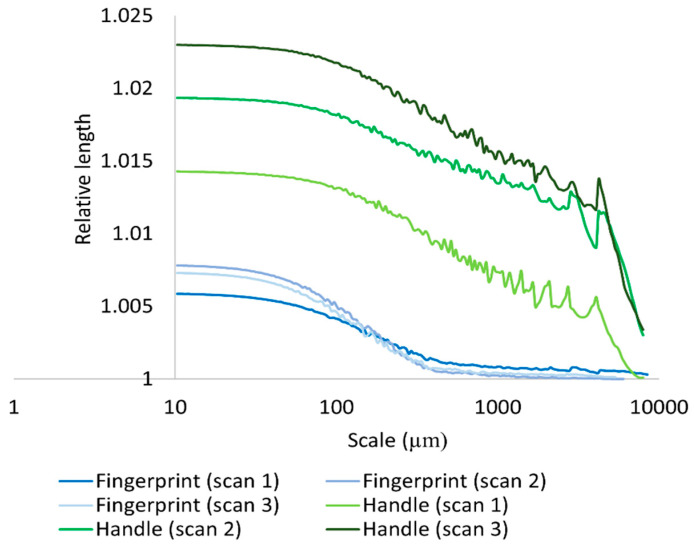
Mean RL for the experimental hardened clay blocks with fingerprints [Fingerprint (scans 1–3)] and the fingerprint on the pottery handle fragment [Handle (scans 1–3)] based on profiles perpendicular to the orientation of the fingerprint ridges and furrows.

In addition to relying on three-dimensional multiscalar areal measurements to mathematically document and discriminate the surfaces of the hardened clay blocks with and without fingerprints and the region on the pottery handle fragment with a fingerprint, we also employed the two-dimensional multiscalar profile measurements (RL and Lsfc). Our results for the mean RL of three profiles taken on each of the measured clay surfaces indicated that mean RL increased with a decreasing scale of measurement and that there were mean RL differences between the surfaces of the hardened clay blocks with and without fingerprints and the region on the pottery handle fragment with a fingerprint over multiple length scales (Figure 22, Figure 23 and Figure 24). Of note, the experimental and archaeological clay surfaces with fingerprints all demonstrated greater mean RL at finer scales than the experimental clay surfaces without prints. Furthermore, the mean RL for the fingerprint on the pottery handle fragment was greater than the mean RL for the surfaces with fingerprints on the hardened clay blocks (Figure 24).

**Figure 25:**
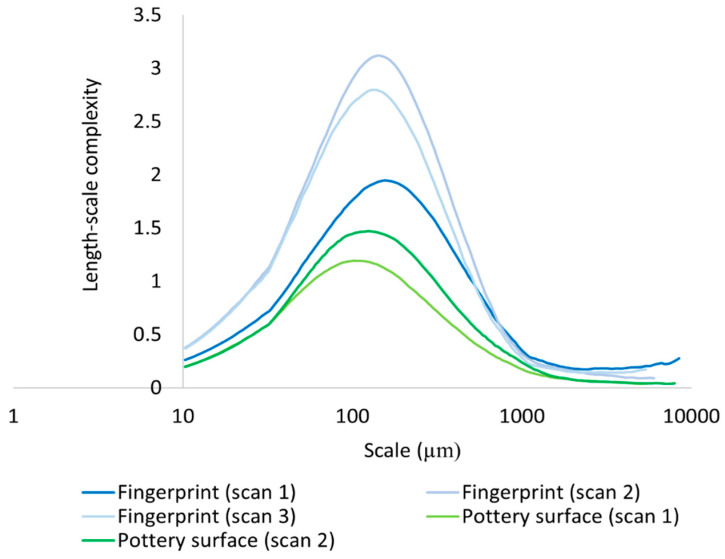
Mean Lsfc for the experimental hardened clay blocks with [Fingerprint (scans 1–3)] and without [Pottery surface (scans 1–2)] fingerprints based on profiles perpendicular to the orientation of the fingerprint ridges and furrows.

**Figure 26:**
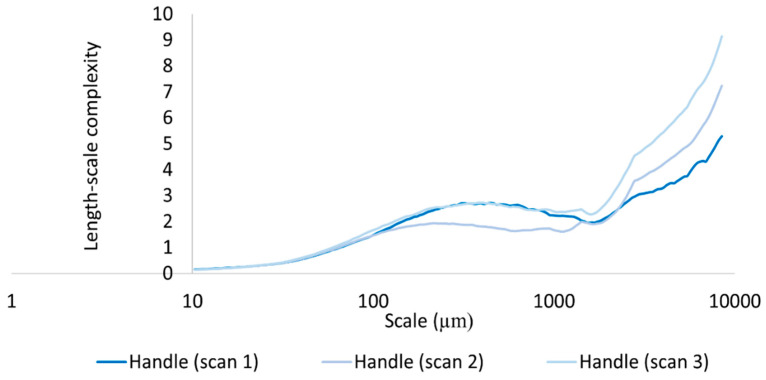
Mean Lsfc for the fingerprint on the pottery handle fragment [Handle (scans 1–3)] based on profiles perpendicular to the orientation of the fingerprint ridges and furrows.

**Figure 27:**
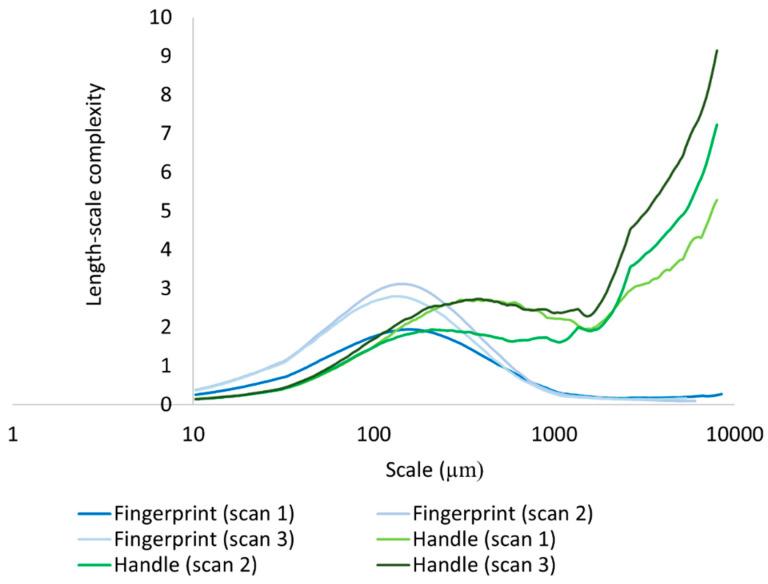
Mean Lsfc for the experimental hardened clay blocks with fingerprints [Fingerprint (scans 1–3)] and the fingerprint on the pottery handle fragment [Handle (scans 1–3)] based on profiles perpendicular to the orientation of the fingerprint ridges and furrows.

We calculated differences in mean Lsfc for all measured surfaces on the hardened clay blocks with and without fingerprints and the fingerprint on the pottery handle fragment over multiple length scales (Figure 25, Figure 26 and Figure 27). At both coarse and fine scales of measurement, mean Lsfc was greater for the surfaces with fingerprints on the hardened clay blocks and the pottery handle fragment than the hardened clay block surfaces without fingerprints. At the finest length scales below 100 µm, mean Lsfc for the profiles from the three measurements of the fingerprint on the pottery handle fragment converge, indicating that, at this scale, the three measurements of the same fingerprint cannot be discriminated (Figure 27).

**Figure 28:**
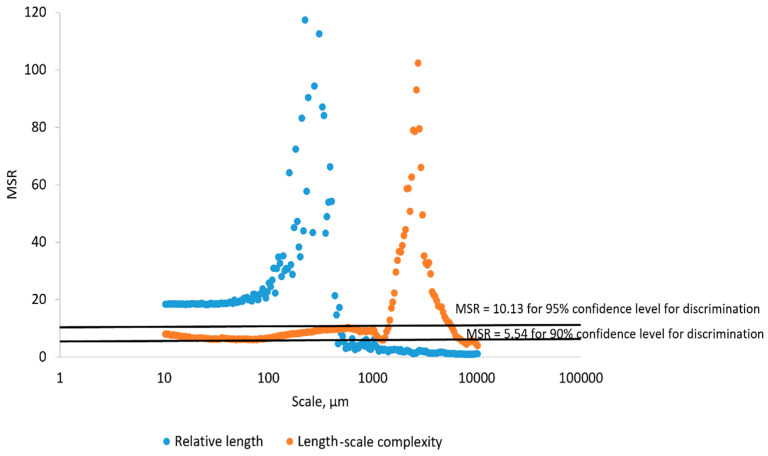
MSRs of RL and Lsfc for discriminating the hardened clay blocks with [Fingerprint (scans 1–3)] and without [Potter surface (scans 1–2)] fingerprints based on profiles perpendicular to the orientation of the fingerprint ridges and furrows. The two horizontal lines denote the minimum MSR of 90% and 95% confidence levels where discrimination is possible.

In order to determine if the differences in mean RL and mean Lsfc for the surfaces of the hardened clay blocks without fingerprints and those with fingerprints were statistically significant, we calculated MSRs with confidence level thresholds set at 90% and 95% (Figure 28). Using MSRs of mean RL, our results could discriminate the different surfaces above the 90% or 95% confidence levels at fine scales below 1000 µm. In contrast, MSRs of mean Lsfc were only capable of discriminating these same surfaces at scales between 10,000 and 1000 µm above the 90% and 95% confidence levels and at finer scales below 1000 µm above the 90% confidence threshold.

**Figure 29:**
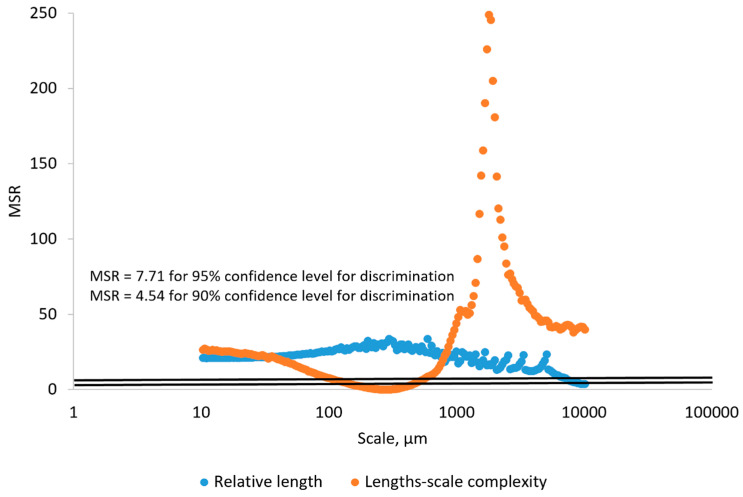
MSRs of mean RL and Lsfc for discriminating the experimental hardened clay blocks with fingerprints [Fingerprint (scans 1–3)] and the fingerprint on the pottery handle fragment [Handle (scans 1–3)] based on profiles perpendicular to the orientation of the fingerprint ridges and furrows. The two horizontal lines denote the minimum MSR of 90% and 95% confidence levels where discrimination is possible.

For the surfaces on the hardened clay blocks with fingerprints and the pottery handle fragment with the fingerprint, our results for the MSRs of mean RL demonstrated discrimination was possible at almost any scale above the 90% and 95% confidence levels, except for very coarse scales around 10,000 µm (Figure 29). However, based on our MSR calculations, the discrimination of mean Lsfc for the hardened clay blocks with fingerprints and the pottery handle fragment with the fingerprint occurred at finer scales slightly below 100 µm and above approximately 800 µm for the 90% and 95% confidence levels (Figure 29).

## 4. Discussion

Based on our results from this first experiment documenting surface topography on hardened clay, the GelSight Max instrument may prove to be a reliable tool for measuring pottery surfaces and analyzing fingerprints that have survived on archaeological ceramic artifacts, but more testing of this technology is required. Based on the 2D and 3D height maps produced from the impressions in the elastomer gel membrane in this study, this instrument could accurately document surface details of the fingerprints, notably the ridges and furrows. Much like the results from a previous study on stone tool surfaces and microwear [6], it is possible to calculate surface roughness using conventional height and hybrid parameters, as well as R^2^ and Das, based on the height maps; however, the experimental and archaeological hardened clay surfaces could not be discriminated based on surface roughness in some instances. In addition, the height maps produced using the instrument allowed us to both characterize and discriminate the surfaces of the hardened clay blocks with and without fingerprints, as well as the surfaces of the hardened clay with fingerprints and the fingerprint preserved on an archaeological pottery handle fragment using the four different multiscalar parameters (Srel, Asfc, RL, and Lsfc). Our results demonstrated that these parameters varied over multiple measurement scales. Both Srel and RL increased as the scale of measurement decreased and both Asfc and Lsfc varied over different scale ranges [76].

In this study, an interesting phenomenon that we noted in the surface characterization data for both Srel and RL for the measurements of the fingerprint on the pottery handle fragment was similarly observed in another experiment using the GelSight Max instrument on stone tool surfaces [6]. In both the current study and the previous one, the plot lines level off below 1000 µm^2^ in the Srel graphs (Figure 14 and Figure 17) and below 100 µm in the RL graphs (Figure 23 and Figure 24). We contend that this ‘flatlining’ phenomenon may be explained by the instrument’s resolution that cannot reliably capture the hardened clay surfaces (and stone tool surfaces) below a certain scale of measurement. As also suggested in the other study [6], this ‘flatlining’ may be due to the fact that the gel membrane on the GelSight Max instrument’s probe cannot accurately document surfaces’ features at fine scales below these thresholds. This may be due to the instrument’s measurable surface roughness and z-axis/height resolution (see Table 2). Both of these limitations may play a role in the ‘flatlining’ phenomenon discussed above. Moreover, the instrument’s gel membrane may be incapable of accurately measuring some fine scale surface features, which may be caused by physical limits of the elastomer gel membrane itself. The gel membrane may be incapable of conforming well enough to some fine scale features on surfaces that are pressed into it to enable measurement by the LED light on the reflective backing of the membrane. Primary reasons for this may include both the thickness and elasticity of the gel membrane and the particular shapes of the micro- or nanoscale surface features pressed into the membrane. It is also worth noting that additional challenges to the quantification of fingerprints on pottery surfaces may be due to the conditions associated with the print impressions themselves, specifically the clay composition of the surfaces, clay firing techniques, and clay shrinkage resulting from firing [32,38].

Two notable benefits of the GelSight Max instrument are its ease of operation and its portability. It is a self-contained measurement system with a rapid surface capture speed (0.5 s) per measurement. The instrument operates on its own battery power and weighs less than 1 kg. As such, it is ideal for analyzing artifacts in a variety of locations, including field studies, museums, universities, art dealers, auction houses, and private collections. Additional benefits include the instrument’s reliance on the elastomer tactile sensor, which eliminates any working distance issues [78], and its bidirectional reflectance distribution function (BRDF) (i.e., the LED array that illuminates the reflective membrane on the back of the elastomer gel pad), which allows the instrument to function in all lighting situations [42,44,79].

## 5. Conclusions

The mathematical micro- and nanoscale documentation of surfaces has become an important aspect of archaeological research over the last couple of decades [46]. Therefore, the adoption of new technologies and the development of new techniques to document surfaces on artifacts and ecofacts are the primary impetus for further testing to determine what they can and cannot do. Our research in this study tested the GelSight Max instrument’s ability to mathematically document the surfaces of hardened clay with and without fingerprints and a fingerprint impression that survived on an archaeological pottery handle fragment. The surface data acquired using this instrument were used to calculate different height and hybrid parameters, in addition to the fractal dimension, and four multiscale surface roughness algorithms. The results of this experiment represent just the second demonstration of the potential value of this instrument to archaeologists [6], but they should be considered preliminary as the overall sample size in the current study is small. More impressions of fingerprints preserved in hardened clay recovered from archaeological deposits need to be documented using the GelSight Max instrument and their surface textures calculated using the same parameters employed in this study. With a larger sample size of fingerprints from the past, the method presented in this paper can be further evaluated in terms of its applicability to archaeological ceramics. However, fingerprints on archaeological pots, figurines, or other hardened clay surfaces are quite rare in the archaeological record and the quality of the preservation of the prints themselves varies tremendously. Notably, a print has limited value if the surficial characteristics of the ridges and furrows are not preserved well enough. As with any new technology, more testing would be valuable to determine the range of applications of the GelSight Max instrument to better understand the material culture of the past.

## Figures and Tables

**Table 1:** Surface roughness/texture parameters used in this study.

Height Parameters	Descriptions (Units of Measurement)
Sa	Arithmetical mean height (µm)
Sp	Maximum peak height (µm)
Sq	Root mean square of height (µm)
Sku	Kurtosis–sharpness of roughness profile
Ssk	Skewness–degree of bias of roughness shape (asperity)
Sv	Maximum pit depth (µm)
Sz	Maximum height of scale-limited surface (µm)
**Hybrid parameters**	**Descriptions (units of measurement)**
Sdq	Root mean square gradient
Sdr	Developed interfacial area ratio (%)
**Multiscale geometric parameters**	**Descriptions (units of measurement)**
Das	Fractal dimension
Asfc	Area-scale complexity (µm^2^)
Lsfc	Length-scale complexity (µm)
RL	Relative length (µm)
Srel	Relative area (µm^2^)

**Table 2:** Specifications for the GelSight Max measurement system (from the official GelSight manufacturer’s website—https://www.gelsight.com).

Parameter	Value and Unit of Measurement
Field of view	14.6 mm × 8.3 mm
Surface roughness range	0.2–20 μm
x-y plane accuracy	3 μm + 0.2%
z-axis/height (1–50 μm) accuracy	0.3 μm + 4%
Capture speed	0.5 ms

**Table 3:** Surfaces measured using the GelSight Max with their corresponding scan designations.

**Hardened Clay—no fingerprint (experimental)**	**GelSight Max scan designation**
Block 1	Pottery surface (scan 1)
Block 2	Pottery surface (scan 2)
**Hardened clay—fingerprint (experimental)**	**GelSight Max scan designation**
Individual 1—right thumbprint 1	Fingerprint (scan 1)
Individual 2—right thumbprint 1a	Fingerprint (scan 2)
Individual 2—right thumbprint 1b	Fingerprint (scan 3)
**Pottery handle fragment—fingerprint (archaeological)**	**GelSight Max scan designation**
Pottery handle fragment 1—print 1a	Handle (scan 1)
Pottery handle fragment 1—print 1b	Handle (scan 2)
Pottery handle fragment 1—print 1c	Handle (scan 3)

**Table 4:** Mean conventional parameters (height and hybrid) with standard deviations calculated from the hardened clay blocks’ and pottery handle fragment’s surfaces.

	Pottery Surface (Scan 1) Pottery Surface (Scan 2)	Fingerprint (Scan 1) Fingerprint (Scan 2) Fingerprint (Scan 3)	Handle (Scan 1) Handle (Scan 2) Handle (Scan 3)
Sa (µm) [StDev]	22.627 [5.907]	46.678 [15.296]	464.167 [30.059]
Sq (µm) [StDev]	28.038 [7.832]	58.135 [20.524]	548.299 [38.113]
Ssk [StDev]	0.305 [0.714]	0.167 [0.477]	−0.637 [0.136]
Sku [StDev]	3.110 [0.255]	2.823 [0.230]	2.278 [0.184]
Sp (µm) [StDev]	92.205 [46.280]	185.407 [84.956]	753.868 [13.088]
Sv (µm) [StDev]	80.950 [0.141]	153.617 [67.920]	1324.887 [112.948]
Sz (µm) [StDev]	173.155 [46.266]	339.023 [145.537]	2078.756 [115.536]
Sdq [StDev]	0.125 [0.007]	0.153 [0.006]	0.493 [0.057]
Sdr (%) [StDev]	0.805 [0.078]	1.170 [0.053]	10.743 [2.152]

**Table 5:** Mean multiscalar parameters with standard deviations calculated from the hardened clay blocks’ and pottery handle fragment’s surfaces.

	Pottery Surface (Scan 1) Pottery Surface (Scan 2)	Fingerprint (Scan 1) Fingerprint (Scan 2) Fingerprint (Scan 3)	Handle (Scan 1) Handle (Scan 2) Handle (Scan 3)
R^2^ [StDev]	0.9985 [0.0006]	0.9863 [0.0221]	0.9206 [0.1173]
Das [StDev]	2.0009 [0.0002]	2.0018 [0.0003]	2.0501 [0.0647]

## Data Availability

The original contributions presented in the study are included in the article; further inquiries can be directed to the corresponding author.
